# Air pollution events from forest fires and emergency department attendances in Sydney, Australia 1996–2007: a case-crossover analysis

**DOI:** 10.1186/1476-069X-13-105

**Published:** 2014-12-10

**Authors:** Fay H Johnston, Stuart Purdie, Bin Jalaludin, Kara L Martin, Sarah B Henderson, Geoffrey G Morgan

**Affiliations:** Menzies Research Institute Tasmania, University of Tasmania, Private Bag 23, Hobart, Tasmania Australia; Biostatistical Officer Training Program, NSW Ministry of Health, Sydney, New South Wales Australia; Ingham Institute of Applied Medical Research, University of New South Wales, Sydney, NSW Australia; Epidemiology Group, Healthy People and Places Unit, South Western Sydney Local Health District, Sydney, NSW Australia; Cancer Council of Victoria, Melbourne, VIC Australia; School of Plant Science, University of Tasmania, Hobart, Tasmania Australia; British Colombia Centre for Disease Control, Vancouver, British Columbia Canada; University Centre for Rural Health – North Coast, University of Sydney, Lismore, New South Wales Australia; Northern New South Wales Local Health District, Lismore, Australia

**Keywords:** Forest fires, Air pollution, Emergency departments attendances, Case crossover, Respiratory, Cardiovascular

## Abstract

**Background:**

Severe air pollution generated by forest fires is becoming an increasingly frequent public health management problem. We measured the association between forest fire smoke events and hospital emergency department (ED) attendances in Sydney from 1996–2007.

**Methods:**

A smoke event occurred when forest fires caused the daily citywide average concentration of particulate matter (PM_10_ or PM_2.5_) to exceed the 99th percentile of the entire study period. We used a time-stratified case-crossover design and conditional logistic regression models adjusted for meteorology, influenza epidemics, and holidays to estimate odds ratios (OR) and 95% confidence intervals (CI) for ED attendances on event days compared with non-event days for all non-trauma ED attendances and selected cardiorespiratory conditions.

**Results:**

The 46 validated fire smoke event days during the study period were associated with same day increases in ED attendances for all non-trauma conditions (1.03, 95% CI 1.02, 1.04), respiratory conditions (OR 1.07, 95% CI 1.04, 1.10), asthma (OR 1.23, 95% CI 1.15, 1.30), and chronic obstructive pulmonary disease (OR 1.12, 95% CI 1.02, 1.24). Positive associations persisted for one to three days after the event. Ischaemic heart disease ED attendances were increased at a lag of two days (OR 1.07, 95% CI 1.01, 1.15) while arrhythmias had an inverse association at a lag of two days (OR 0.91, 95% CI 0.83, 0.99). In age-specific analyses, no associations present in children less than 15 years of age for any outcome, although a non-significant trend towards a positive association was seen with childhood asthma. A further association between smoke event and heart failure attendances was present for the 15–65 year age group, but not older adults at a lag of two days (OR 1.37 95% CI 1.05, 1.78).

**Conclusion:**

Smoke events were associated with an immediate increase in presentations for respiratory conditions and a lagged increase in attendances for ischaemic heart disease and heart failure. Respiratory impacts were either absent or considerably attenuated in those <15 years. Similar to previous studies we found inconsistent associations between fire smoke and cardiovascular diseases. Better characterisation of the spectrum of population health risks is needed to guide public heath responses to severe smoke events as this exposure becomes increasingly common with global climate change

**Electronic supplementary material:**

The online version of this article (doi:10.1186/1476-069X-13-105) contains supplementary material, which is available to authorized users.

## Introduction

Severe air pollution generated by forest fires across the globe is becoming an increasingly frequent public health management problem. As climate change increases the conditions favourable to severe fires [1], populations living in fire prone areas, are expanding, especially at the urban fringes [2] and more deliberate landscape burning is being conducted to protect property and infrastructure from extreme events [3, 4].

There is a comparatively small body of public health evidence specifically addressing the health impacts of severe forest fire smoke events. Public health officials often need to generalise from the wider urban particulate air pollution and health literature to guide major decisions about public health protection (i.e. whether to evacuate a community) in response to severe smoke episodes [5]. However, urban particulate air pollution and forest fire smoke have different compositions, and different durations and severities of exposure. Smoke includes hundreds of aerosolised compounds of both elemental and organic carbon [6, 7], numerous organic and inorganic gases, and other toxins including metals and free radicals [8]. The mixture changes through space and time depending on the combustion conditions, and as compounds react with each other [9].

Forest fire smoke can cause extremely high concentrations of atmospheric particulate matter (PM). However, with the important exception of tropical deforestation and savanna fires, the exposures are generally short lived, commonly from hours to weeks [10]. This makes studying the health impacts of forest fire smoke challenging. It is difficult to detect relatively small public health impacts unless large populations are regularly exposed and there is a reliable method for quantifying that exposure.

There is good evidence that extreme pollution from severe forest fires contributes to excess mortality, hospital admissions, and exacerbations of respiratory illnesses over large demographic areas [11–15]. Most evidence has come from analyses of administrative mortality and hospital admissions data, and the research has largely focussed on respiratory impacts. Contrary to the well-established association between urban particulate air pollution and adverse cardiovascular outcomes, reported associations between forest fire smoke and cardiovascular outcomes have been inconsistent [16]. It is not clear whether the limited evidence for cardiovascular outcomes reflects the limited research in this area, or a true difference between the epidemiologic impacts of urban and forest fire PM.

Hospital emergency department (ED) attendances provide an additional source of information for evaluating the range of health outcomes associated with forest fire smoke. Published studies of emergency department attendances and fire smoke episodes are few in number and have generally been confined to the evaluation of respiratory health outcomes [12, 17, 18]. One notable exception was a study of smoke from a peat bog fire by Rappold et al. [19], which assessed a wide range of outcomes and identified associations between smoke periods and attendances for heart failure, and several respiratory health outcomes.

A previously validated database of episodes of poor air quality due to forest fire smoke in Sydney, Australia for the period 1996–2007 has been used to assess the impacts of forest fire smoke on mortality and hospital admissions [13, 16, 20]. Here we use the same exposure data to study ED attendances for both respiratory and cardiovascular conditions.

## Methods

### Setting

Sydney lies on a coastal lowland plain between the Pacific Ocean and elevated sandstone tablelands largely covered with thick eucalypt forests in the state of New South Wales (NSW), Australia. The main sources of background PM include motor vehicle and industrial emissions, domestic wood smoke in winter, and crustal particles [21]. The vast majority of fire smoke is derived from planned and wild fires in the eucalypt forests in the Blue Mountains to the west of the region [22]. Although fire activity is highly variable, Sydney is affected by severe pollution events from fires for an average of 4–5 days each year [20].

### Study population

The population of Sydney was 4.06 million at the 2001 census [23]. Participants were identified from the Emergency Department Data Collection (EDDC) maintained by the NSW Ministry of Health for the period 1 July 1996 to 30 June 2007. Records were selected for patients residing in statistical local areas (SLAs) corresponding with the Sydney metropolitan area. The patient’s SLA of residence was absent from approximately 80% of records a four-month period during 2002 and 2003. We used the postcode of residence to derive the SLA in these cases. The details of this process are presented in Additional file 1. We excluded presentations coded as planned (e.g. for an elective procedure), planned return visits (e.g. for results), and unplanned return visits for ongoing management of the same condition. In Australia, the usual practice is for acute health problems to be evaluated in emergency departments before being admitted, even if they have been referred by an outpatient service or private medical practitioner.

### Outcome data

The reason for attendance at ED was based on the provisional emergency department diagnosis, coded using the World Health Organisation's International Classification of Diseases (ICD) [24]. Coding changed from the 9^th^ to the 10^th^ revision of the ICD during the study period. We followed national health data mapping protocols to ensure consistent classification between these two versions [25]. We investigated ED presentations for the following conditions: all non-trauma; all respiratory; asthma; chronic obstructive pulmonary disease (COPD); pneumonia and bronchitis; all cardiovascular; ischemic heart disease; arrhythmias; cardiac failure; and cerebrovascular diseases (Table 1).

**Table 1:** **ICD codes used to define reason for presentation to ED**

Reason for presentation	ICD-9	ICD-10-AM
All non-trauma causes	001-799	A00-R99
Respiratory diseases (all)	460-519	J00-J99 (excluding J95.4 to J95.9), R09.1, R09.8
Asthma	493	J45-J46,
Chronic obstructive pulmonary disease (COPD)	490-492, 494-496	J40-J44, J47, J67
Pneumonia or acute bronchitis	466, 480-486	J12-J17, J18.0, J18.1, J18.8, J18.9, J20, J21
Cardiovascular diseases (all)	390-459	I00-I99 (excluding I67.3, I68.0, I88, I97.8, I97.9, I98.0), G45 (excluding G45.3), G46, M30, M31, R58
Ischemic heart diseases (IHD)	410-413	I20-I25
Arrhythmias	427	I46-I49
Cardiac failure (CF)	428	I50
Cerebrovascular diseases	430-438	I60-I69 (excluding I67.0, I67.3, I68.0), G45 (excluding G45.3), G46

### Exposure data

A database of historical vegetation fire smoke events for Sydney was compiled and validated as part of a previous study and the methods are reported separately [20]. In summary, smoke events were defined as days when the citywide average of PM concentrations (measured as either PM_10_ or PM_2.5_) exceeded the 99^th^ percentile of the entire time series (47 μg/m^3^ for PM_10_ and 27 μg/m^3^ for PM_2.5_) and the elevated PM could be attributed to smoke from wild or prescribed forest fires. The citywide averages were derived from seven monitoring stations in the Sydney metropolitan area. Evidence that poor air quality was due to forest fire smoke was sought from several sources. These included media reports of bushfires and smoke haze, government land management agency records of bushfires and planned burns, and images from the Moderate Resolution Imaging Spectroradiometer (MODIS) aboard NASA’s Terra and Aqua satellites which show the location of active fires and smoke plumes. Prior to the launch of these satellites in 2000 an earlier platform, the total ozone mapping spectrometer, was used to identify if smoke and dust episodes affecting on days of high particle pollution. During the study period 95% of exceedances could be explained by forest fire smoke episodes. For full details see Johnston et al. 2011 [20]. Two days in which the elevated PM was attributed to dust storms were excluded.

The cut points for the 99^th^ percentile were similar to 24 hour average air quality standards for PM in Australia, which are currently 50 μg/m^3^ for PM_10_ and 25 μg/m^3^ for PM_2.5_ [26]. Although the study population has been exposed to further forest fire smoke episodes since 2007, those events have not been validated using the same rigorous methods employed for the 1996–2007 database so they were not included in these analyses.

### Other covariates

The Australian Bureau of Meteorology provided daily average ambient temperature and dew point as a measure of humidity. Data from weather stations within 50 kilometres of the population-weighted centre of the city were used to calculate daily averages for the study population of Sydney [27]. Influenza epidemics were identified as days on which the number of admissions to hospital for influenza exceeded the 90^th^ percentile of all daily counts over the study period. Public holidays and school holidays for NSW were obtained from published calendars [28].

### Statistical analysis

We used a time-stratified case-crossover design [29, 30]. Each patient presenting to ED for a specific condition was considered as a case on the date of attendance and as a control on selected other days in the same calendar month. The effect of smoke events was compared between case and control days. Control days were matched on year, month, and day-of-week to control for the potentially confounding effects of day-of-week, seasonal cycles, and long term trends in ED attendances [30]. We estimated the association between smoke events and the risk of ED attendances using conditional logistic regression models adjusted for the potential confounding effects of temperature and dew point (both same-day and the average for the previous three days), influenza epidemics, and public holidays. The effect of school holidays was also included in the modelling of ED presentations in children, because asthma and respiratory infections have been shown to increase in association with school terms [31].

The relationship between number of ED presentations and the temperature measures was non-linear. Natural cubic splines were fitted to temperature and dew point using four degrees of freedom for temperature splines (same day and lagged) and three degrees of freedom for the dew point splines (same day and lagged). The sensitivity of our results to the number of degrees of freedom used for these covariates was evaluated. See Additional file 1 for details. All analyses were performed using the R statistical software using the packages ‘splines’ and ‘survival’ (version 2.13.1) [32].

### Ethical approval

This study was approved by the Tasmanian Human research Ethics Committee (H0010047) and the Human Research Ethics Committee of the Australian National University (2008/199).

## Results

**Figure 1:**
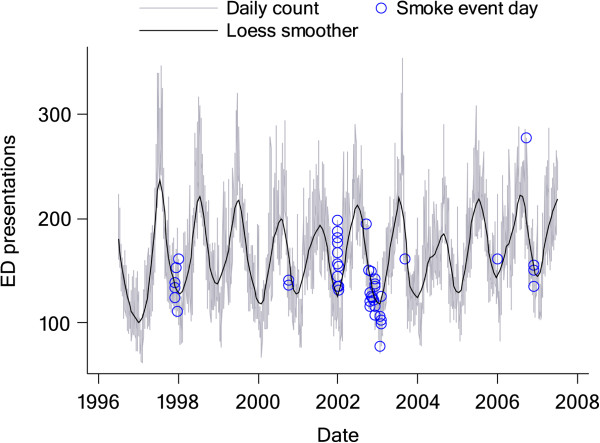
**Daily counts of emergency department attendances for respiratory conditions (grey line) with smoother fitted by locally weighted polynomial regression (black line).** The circles indicate counts on smoke event days for Sydney, 1 July 1996 to 30 June 2007.

Over the 11-year period from 1 July 1996 to 30 June 2007, there were 46 smoke event days. Two of these event days were due to planned vegetation fires and the remaining 44 were due to wildfires. Mean PM_10_ and PM_2.5_ were 60.5 and 39.1 μg/m^3^ on smoke days compared with 17.8 and 9.9 μg/m^3^ on non-smoke days, respectively. On average, days affected by forest fire smoke were warmer than other days, while humidity was similar on smoke days and non-smoke days (Table 2). Over the same period there were more than 630,000 ED presentations by Sydney residents for respiratory conditions and close to 370,000 presentations for cardiovascular conditions (Table 3). Children less than 15 years of age accounted for 25% of the total number of attendances and for about half of all attendances for respiratory conditions (all), asthma, and pneumonia or acute bronchitis. As expected, very few children less than 15 years were diagnosed with COPD or with any of the cardiovascular conditions that were assessed (Table 3). There was wide daily and seasonal variability in counts of ED presentations for all respiratory conditions (Figure 1).

**Table 2:** **Mean and range of daily average readings for particulate matter and temperature across Sydney, Australia over the period 1 July 1996 to 30 June 2007**

	Non-smoke event days (n = 3971)		Smoke event days (n = 46)	
Measure	Mean	Range	Mean	Range
PM_10_ (μg/m^3^)	17.8	(4.0, 199.2)	60.5	(32.0 - 114.8)
PM_25_ (μg/m^3^)	9.9	(2.1, 46.7)	39.1	(14.6 - 100.2)
Temperature (°C)	18.4	(8.7, 33.9)	24.6	(14.5 - 32.8)
Dew point (°C)	10.7	(-4.3, 21.9)	10.7	(1.0 - 18.3)

**Table 3:** **Mean and range of daily counts of emergency presentations to emergency departments by Sydney residents over the period 1 July 1996 to 30 June 2007 for respiratory and cardiovascular conditions**

	Attendances	Daily counts		Age profile		
Principal reason for attendance	Total	Mean	Range	< 15 years	15-64 years	65 + years
**All non-trauma attendances**	**4,655,639**	**1159**	**(728, 1782)**	**25%**	**51%**	**23%**
**All respiratory conditions**	**663,333**	**165**	**(62, 354)**	**52%**	**29%**	**19%**
Asthma	132,634	37	(6, 127)	56%	37%	7%
COPD	50,194	12	(1, 33)	1%	23%	75%
Pneumonia or acute bronchitis	130,915	33	(3, 88)	49%	22%	29%
**All cardiovascular conditions**	**368,423**	**92**	**(50, 142)**	**1%**	**39%**	**60%**
Ischemic heart disease	101,742	25	(5, 54)	0%	43%	57%
Arrhythmias	64,398	16	(2, 36)	1%	42%	57%
Cerebrovascular disease	63,564	16	(4, 34)	0%	26%	73%
Cardiac failure	48,126	12	(1, 34)	0%	13%	87%

Smoke event days were associated with increased risk of ED presentations for all non-trauma conditions, all respiratory conditions, asthma, and COPD at different lags. Overall and age-specific results are shown in Table 4.

**Table 4:** **Estimated odds ratios (OR) for the associations between smoke event days and presentations to emergency departments for all non trauma, respiratory and cardiovascular conditions by age-group, Sydney, 1 July 1996 and 30 June 2007**

		All ages		Age < 15 years		15-64 years		Age 65 + years	
Condition	Lag	OR	95% CI	OR	95% CI	OR	95% CI	OR	95% CI
All non trauma	0	**1.03**	**(1.02-1.04)**	1.01	(0.99-1.04)	**1.04**	**(1.02-1.05)**	**1.02**	**(1.00-1.04)**
	1	**1.02**	**(1.01-1.03)**	1.00	(0.98-1.02)	**1.03**	**(1.01-1.04)**	**1.02**	**(1.00-1.04)**
	2	**1.02**	**(1.01-1.03)**	0.98	(0.96-1.00)	**1.03**	**(1.02-1.05)**	**1.02**	**(1.00-1.04)**
	3	**1.01**	**(1.00-1.02)**	0.99	(0.97-1.01)	**1.02**	**(1.00-1.03)**	**1.02**	**(1.00-1.04)**
All Resp	0	**1.07**	**(1.04-1.10)**	1.01	(0.97-1.06)	**1.16**	**(1.10-1.22)**	1.06	(0.99-1.13)
	1	**1.05**	**(1.02-1.08)**	1.00	(0.96-1.05)	**1.11**	**(1.06-1.17)**	1.05	(0.99-1.12)
	2	1.00	(0.97-1.03)	0.94	(0.90-0.98)	**1.06**	**(1.01-1.12)**	1.03	(0.97-1.10)
	3	1.01	(0.98-1.04)	0.97	(0.93-1.01)	1.04	(0.99-1.10)	1.05	(0.98-1.12)
Asthma	0	**1.23**	**(1.15-1.30)**	1.06	(0.97-1.17)	**1.38**	**(1.26-1.52)**	**1.45**	**(1.16-1.79)**
	1	**1.18**	**(1.11-1.26)**	1.05	(0.96-1.15)	**1.30**	**(1.18-1.43)**	**1.35**	**(1.09-1.67)**
	2	**1.14**	**(1.07-1.22)**	0.97	(0.89-1.07)	**1.32**	**(1.20-1.45)**	**1.37**	**(1.11-1.69)**
	3	**1.10**	**(1.03-1.17)**	1.00	(0.91-1.09)	**1.18**	**(1.07-1.31)**	**1.35**	**(1.10-1.66)**
COPD	0	**1.12**	**(1.02-1.24)**	*	*	**1.23**	**(1.01-1.50)**	1.08	(0.96-1.21)
	1	1.03	(0.93-1.14)	*	*	1.00	(0.81-1.24)	1.04	(0.93-1.17)
	2	0.96	(0.87-1.06)	*	*	0.96	(0.78-1.18)	0.97	(0.86-1.09)
	3	1.03	(0.93-1.14)	*	*	1.02	(0.83-1.25)	1.03	(0.92-1.16)
Pneumonia/bronchitis	0	1.02	(0.95-1.10)	0.96	(0.85-1.07)	1.09	(0.95-1.25)	1.06	(0.94-1.19)
	1	1.00	(0.93-1.07)	0.97	(0.87-1.09)	0.89	(0.78-1.03)	1.11	(0.99-1.25)
	2	1.02	(0.95-1.10)	1.05	(0.94-1.18)	0.96	(0.84-1.11)	1.04	(0.92-1.18)
	3	1.00	(0.93-1.07)	1.01	(0.90-1.13)	0.95	(0.83-1.10)	1.02	(0.90-1.16)
All cardio-vascular	0	1.00	(0.96-1.04)	0.86	(0.54-1.35)	0.98	(0.93-1.04)	1.01	(0.97-1.06)
	1	0.99	(0.95-1.03)	1.14	(0.76-1.73)	0.96	(0.90-1.01)	1.01	(0.96-1.06)
	2	1.03	(0.99-1.06)	0.92	(0.57-1.49)	1.03	(0.97-1.09)	1.03	(0.98-1.08)
	3	0.99	(0.96-1.03)	0.85	(0.52-1.39)	0.99	(0.93-1.04)	0.99	(0.95-1.04)
IHD	0	0.99	(0.93-1.06)	*	*	0.94	(0.84-1.04)	1.03	(0.95-1.13)
	1	1.01	(0.95-1.08)	*	*	0.96	(0.86-1.07)	1.05	(0.96-1.15)
	2	**1.07**	**(1.00-1.15)**	*	*	1.02	(0.92-1.14)	**1.11**	**(1.02-1.21)**
	3	0.96	(0.90-1.03)	*	*	0.94	(0.84-1.04)	0.98	(0.90-1.08)
Arrhythmias	0	0.97	(0.89-1.06)	*	*	1.01	(0.89-1.16)	0.94	(0.84-1.06)
	1	**0.91**	**(0.83-0.99)**	*	*	**0.86**	**(0.75-0.99)**	0.94	(0.84-1.06)
	2	0.93	(0.86-1.02)	*	*	0.95	(0.84-1.09)	0.92	(0.82-1.03)
	3	0.94	(0.86-1.03)	*	*	0.91	(0.80-1.04)	0.96	(0.85-1.08)
Cerebrovasc	0	0.99	(0.91-1.08)	*	*	1.00	(0.84-1.18)	0.99	(0.90-1.10)
	1	0.99	(0.91-1.08)	*	*	0.84	(0.70-1.00)	1.05	(0.95-1.16)
	2	0.97	(0.89-1.06)	*	*	0.94	(0.79-1.11)	0.99	(0.89-1.10)
	3	1.01	(0.93-1.10)	*	*	1.10	(0.94-1.29)	0.98	(0.89-1.09)
Cardiac failure	0	1.05	(0.95-1.17)	*	*	1.16	(0.87-1.55)	1.03	(0.92-1.16)
	1	0.95	(0.85-1.05)	*	*	0.96	(0.72-1.29)	0.94	(0.84-1.06)
	2	1.04	(0.94-1.16)	*	*	**1.37**	**(1.05-1.78)**	1.00	(0.89-1.11)
	3	1.02	(0.91-1.13)	*	*	1.11	(0.84-1.46)	1.00	(0.89-1.12)

Bold p < 0.05 *insufficient numbers for analysis. COPD = Chronic obstructive pulmonary disease, IHD = ischaemic heart disease, Cerebrovasc = Cerebrovascular disease.

In children less than 15 years of age there was no association between smoke events and ED attendances for all non-trauma conditions or all respiratory conditions. For asthma, positive associations were present for lags 0 and 1 but these did not attain statistical significance. This was in marked contrast with asthma presentations in adults which were increased across all lags by approximately 30 percent.

There was a non-significant trend towards an association with all cardiovascular conditions at a lag of two days and a significant association with attendances for ischemic heart disease at the same lag that was driven by the 65+ age group. However, the point estimates were inconsistent across the four tested lags. No associations were observed with cardiac failure, other than for the 15-64 year age group at a lag of 2 days. There was an inverse association with at arrhythmias a lag of one day, with point estimates consistent across all lags.

Point estimates were robust to two decimal places in sensitivity analyses conducted to test the influence of using a range of degrees of freedom for the temperature splines and for including and excluding cases with imputed area of residence (see Additional file 1).

## Discussion

We found associations between severe smoke episodes from forest fires and the ED presentations for all non-traumatic outcomes, all respiratory outcomes, asthma, COPD, and ischemic heart disease. The main strengths of this study were the long time period that included almost 50 previously-validated smoke event days [20], the large size of the population and the wide range of ED health outcomes available for analysis. Further, our approach to the analysis of ED presentations was consistent with previous analyses of deaths and admissions to hospital for the same study population [13, 16]. A generally consistent pattern of results from three independent datasets increases the confidence in the findings.

Limitations of the study included the use of citywide averages in defining smoke events, which precluded any assessment of their spatial variation and introduced exposure misclassification that would reduce the ability to detect an association if present [33].

During the study period approximately 80% of presentations to NSW public EDs were captured by the EDDC [34]. If people affected by a smoke event attended an ED that did not contribute to this data collection, our results would have been biased towards a conclusion of no effect. Conversely, if an obvious smoke haze made people more likely than usual to attend an ED this would have biased our results away from the null.

ED diagnoses are often recorded as general signs and symptoms (i.e. ICD-9: 780–789; ICD-10: R00-R69) rather than specific diagnoses [34]. This was true for 22% of the cases in our dataset, and we classified them as non-trauma in our analyses. More specifically classifying selected symptoms (e.g. ‘abnormalities of heart beat’ as ‘cardiovascular (all)’, or ‘abnormalities of breathing’ as ‘respiratory (all)’ may have improved the precision of our estimates for those outcomes.

Some health records were missing area of residence. However, the analyses covered the entire Sydney metropolitan area, rather than smaller spatial areas, so use of postcodes to estimate residential location was an acceptable alternative. Sensitivity analyses demonstrated that this approach did not appreciably influence our results (see Additional file 1).

It is clear that smoke events have an influence on the overall workload of hospital emergency departments, demonstrated by the rise of between 1-3% that persisted for four days. Our findings of strong associations between fire smoke and ED presentations for both asthma and COPD are consistent with comprehensive reviews of the available evidence [12]. While our results showed larger effect estimates than that reported for asthma and COPD hospital admissions in Australia [16], Rappold et al. [19] found even larger associations for such ED presentations in a large study of a population exposed to peat fire smoke for a three day period. Additionally, they observed associations with pneumonia and bronchitis, which we did not find in this study. Differences between the studies may be attributable to differences between peat and predominantly eucalypt smoke exposure.

We found that the greatest increase in attendances for respiratory conditions occurred in adults rather than children. While younger age is a recognised higher risk group for the impacts of particulate air pollution in general, age-specific studies of forest fire smoke exposure are limited in number. A previous Australian study of people with asthma found that increased respiratory symptoms and medication use in association with bushfire smoke was much greater in adults than in children less than 18 years [35]. Similarly, a large Canadian cohort study found that the associations with physician visits for asthma ocuured predominantly in adults rather than children [36]. A detailed examination of admissions to hospital during wildfire smoke in California found that admissions were increased in the 0–4 year olds but not those aged 5–14 years and that the greatest increase in asthma admissions was in the 65+ age group [11]. The same pattern was observed during an episode of wildfire smoke in New Mexico where the only age group in which increased ED presentations for asthma was 65+ [37]. Our results suggesting that adults are at greater risk for exacerbations of asthma in response to forest fire smoke are thus consistent with the available literature.

Our results for cardiovascular attendances were mixed, which is also consistent with the literature to date. Studies of associations between forest fire smoke and health care attendances or hospital admissions for ischemic heart diseases (which include angina and acute coronary syndromes) have reported statistically significant decreases [38], increases in high risk population subgroups [38, 39], and no associations [11, 16, 40]. The only other published study of ED attendances found an association with the nonspecific symptom of chest pain, and with the diagnosis of heart failure, but not with a diagnosis of myocardial infarction [19]. Dennekamp et al. [41] observed a large rise in out of hospital cardiac arrest, for which ischemic heart disease is a common precipitant, during severe forest fire smoke episodes in Melbourne, Australia.

Our observation of an inverse relationship between fire smoke events and cardiac arrhythmias does not fit with results from previous reports of this outcome. Neither the Rappold et al. (2011) study of ED attendances nor the Delfino et al. [11] and Martin et al. [16] studies of hospital admissions found positive or negative associations with this diagnostic group. A true protective association is biologically unlikely to be the case. Our result could be a chance finding, or it might reflect a rise in pre-hospital adverse cardiac events such as that demonstrated by Dennekamp et al. (2011) in their study of out of hospital cardiac arrests, which could in turn lead to fewer than expected hospital presentations.

People with chronic respiratory conditions appear to be most sensitive to exacerbations by exposure to forest fire smoke. These are common health conditions that affect more than 30% of the Australian population, with asthma alone affecting 10% of the population [42]. Surveillance systems to support advanced notification of anticipated smoke will benefit those at higher risk, particularly those with asthma and COPD, so that action can be taken to reduce exposure, or to ameliorate the clinical consequences of smoke exposure through the use of preventive medication [43].

More evidence is needed to better characterize how forest fire smoke influences the manifestation of cardiovascular diseases at an individual and population level. Heart disease is a major contributor to the global burden of mortality and morbidity [44], and understanding the influences of forest fire smoke on cardiovascular health is important for mitigating its public health impacts.

## Conclusions

Our results confirm the population-wide impact of severe smoke events from wildfires and the particular vulnerability of people with respiratory conditions. Public health protection will be supported by ongoing efforts to slow the progression of climate change, and specific landscape and fuel management interventions to reduce the frequency and intensity of fire. Systematic surveillance of fires and smoke emissions will be useful to provide advanced warnings for people at higher risk to enable them to take effective action such as the timely use of preventive medications [43], or indoor air filters to reduce exposure particulate matter [45].

## Electronic supplementary material

Additional file 1: **Air pollution events from forest fires and emergency department attendances in Sydney: Selection of covariates, model diagnostics and sensitivity analyses.** (DOCX 155 KB)

## Abbreviations

95% CI: 95% confidence intervals
COPD: Chronic obstructive pulmonary disease
ED: Emergency Department
EDDC: Emergency Department Data Collection
ICD: International Classification of Diseases
NSW: New South Wales
OR: Odds ratio
PM: Particulate matter
SLA: Statistical local area.
