# Supplementary material for: Air pollution events from forest fires and emergency department attendances in Sydney, Australia 1996–2007: a case-crossover analysis
Source: Environ Health. 2014 Dec 10;13:105. doi: 10.1186/1476-069X-13-105 (PMC4271508; doi:10.1186/1476-069X-13-105)
Supplement: Supplementary file 1 — Additional file 1: Air pollution events from forest fires and emergency department attendances in Sydney: Selection of covariates, model diagnostics and sensitivity analyses. (DOCX 155 KB) [file 12940_2014_809_MOESM1_ESM.docx]

**Additional file**

## Air pollution events from vegetation fires and their association with emergency department presentations in Sydney, Australia, 1996-2007: a case-crossover analysis: Selection of covariates, model diagnostics and sensitivity analysis of results by selecting the study population

FH Johnston, S Purdie, B Jalaludin, K Martin, SB Henderson, and GG Morgan.

Author contact fay.johnston@utas.edu.au

## Introduction

This document provides more detail on the handling of covariates and the assessment of model diagnostics in the case-crossover analysis. This analysis was performed in R, fitting a conditional logistic regression as a special case of Cox proportional hazards regression (using the coxph function), where the baseline hazard is different for each stratum and the survival time is uniform across all observations. Rather than provide details for all models, we present examples. Most of the results shown below are from the analysis of the association between vegetation fire smoke events and emergency department (ED) presentations for all respiratory conditions. A vegetation fire smoke event is defined as a day with a particulate matter (PM_10_ or PM_2.5_) reading in the top 1% of all readings across the study period and a confirmed vegetation fire affecting the population.

## Covariates

We identified the following potential predictors of presentations to emergency departments for respiratory and cardiovascular conditions: temperature, humidity (indicated by dew point temperature), flu epidemics and public holidays. School holidays may be associated with presentations due to asthma in children.

### Smoothed meteorological data

The relationship between temperature and humidity and number of presentations to ED for respiratory conditions and 'all-causes' were expected to be non-linear. Natural cubic splines can be fitted to temperature variables to describe the non-linear relationships and the spline bases can be modelled instead of temperature itself. In fitting these splines, we need to determine the optimal number of degrees of freedom (ranges over which the cubic functions are applied) to give a well-fitting smooth curve. We experimented by modelling both all-cause presentations and all respiratory presentations on natural cubic splines of temperature and dew point temperature with varying degrees of freedom (up to a maximum of six). We also included indicator variables for flu epidemics and public holidays in the model. We used both Akaike information criterion (AIC) and Bayesian information criterion (BIC) values to decide on the best model (the model with the best balance between explanatory power and simplicity). For both groups of conditions, models with four degrees of freedom for temperature and three degrees of freedom for dew point were close to optimal on both AIC and BIC (Table 1). The same degrees of freedom were applied to splines for the lagged (previous three-day average) temperature and humidity variables.

The full list of covariates used in the modelling of the risk of ED presentations is detailed in Table 2.

**Table 1:** **Five best models of all-cause and all respiratory condition presentations to ED, obtained by varying the degrees of freedom (df) of natural cubic splines fitted to temperature and an indicator of humidity (dew point).**

| **Five best models for all-cause presentations** | | | | |  |  |
| --- | --- | --- | --- | --- | --- | --- |
| Temperature df | Dew point df | AIC | BIC | AIC rank | BIC rank | Sum of ranks |
| 6 | 3 | 79,470,077 | 79,470,163 | 1 | 4 | 5 |
| 5 | 3 | 79,470,079 | 79,470,156 | 2 | 3 | 5 |
| **4** | **3** | **79,470,083** | **79,470,153** | **8** | **1** | **9** |
| 5 | 4 | 79,470,081 | 79,470,166 | 4 | 7 | 11 |
| 6 | 4 | 79,470,079 | 79,470,172 | 3 | 10 | 13 |
|  |  |  |  |  |  |  |
| **Five best models for all respiratory condition presentations** | | | | | | |
| Temperature df | Dew point df | AIC* | BIC | AIC rank | BIC rank | Sum of ranks |
| 4 | 4 | 8,775,197 | 8,775,275 | 4 | 4 | 8 |
| **4** | **3** | **8,775,200** | **8,775,270** | **8** | **1** | **9** |
| 4 | 2 | 8,775,208 | 8,775,271 | 15 | 2 | 17 |
| 4 | 6 | 8,775,193 | 8,775,286 | 2 | 16 | 18 |
| 3 | 4 | 8,775,205 | 8,775,275 | 14 | 5 | 19 |
| * minimum AIC value for all respiratory was 8,775,193 | | | |  |  |  |

**Table 2:** **Covariates used in modelling the risk of ED presentation.**

| Covariate | Variable name | Values |
| --- | --- | --- |
| Extreme smoke event day | lfs_pm99_lag0 | 1, if extreme smoke event day  0, otherwise |
| Natural cubic spline for temperature with 4 degrees of freedom | ns(temperature, df=4) | set of 4 continuous variables |
| Natural cubic spline for humidity with 3 degrees of freedom | ns(dewpt, df=3) | set of 3 continuous variables |
| Natural cubic spline for lagged temperature (previous 3-day average) with 4 degrees of freedom | ns(temperature, df=4) | set of 4 continuous variables |
| Natural cubic spline for lagged humidity (previous 3-day average) with 3 degrees of freedom | ns(dewpt, df=3) | set of 3 continuous variables |
| Influenza epidemic indicator | flu | 1, if NSW hospital admissions for influenza were in the top 10% of daily counts  0, otherwise |
| Public holiday indicator | pubhol | 1, if the day was a public holiday in NSW  0, otherwise |
| School holiday | schoolhol | 1, if the day was a public school holiday in NSW  0, otherwise |

### Example of a fitted model

Table 2 summarises the results of fitting a model to all respiratory presentations to EDs by Sydney residents. The coefficients of the cubic splines were tested jointly using Wald tests. All of the covariates were highly significant and so none were dropped from the model. Coefficients (and their confidence intervals) were exponentiated to obtain odds ratios. In this case, the odds ratio for extreme smoke event days was $e^{0.07}=1.069$, meaning that smoke event days were associated with an increase of 7% in the odds of presenting to ED with a respiratory condition.

**Table 2: Summary of results of conditional logistic regression of ED presentations for respiratory conditions on extreme smoke events, natural cubic splines for: same day average temperature and dew point temperature; average temperature and dew point temperature averaged over the previous three days; flu epidemics and public holidays.**

|  |  |  |  |  | Wald test |  |  |
| --- | --- | --- | --- | --- | --- | --- | --- |
| Covariate | Coefficient | SE(coef) | z | Pr(>\|z\|) | chi-sq | df | P(>chi-sq) |
| Extreme smoke event | 0.07 | 0.015 | 4.5 | <0.01 |  |  |  |
| ns(temperature, df = 4)1 | 0.02 | 0.013 | 1.7 | 0.09 | 106.1 | 4 | <0.01 |
| ns(temperature, df = 4)2 | 0.11 | 0.014 | 7.9 | <0.01 |  |  |  |
| ns(temperature, df = 4)3 | 0.08 | 0.029 | 2.8 | <0.01 |  |  |  |
| ns(temperature, df = 4)4 | 0.07 | 0.023 | 3.0 | <0.01 |  |  |  |
| ns(dewpt, df = 3)1 | 0.00 | 0.010 | -0.2 | 0.84 | 17.4 | 3 | <0.01 |
| ns(dewpt, df = 3)2 | 0.10 | 0.030 | 3.3 | <0.01 |  |  |  |
| ns(dewpt, df = 3)3 | 0.01 | 0.016 | 0.9 | 0.35 |  |  |  |
| ns(temp_lag, df = 4)1 | -0.08 | 0.014 | -6.2 | <0.01 | 89.8 | 4 | <0.01 |
| ns(temp_lag, df = 4)2 | -0.07 | 0.014 | -4.7 | <0.01 |  |  |  |
| ns(temp_lag, df = 4)3 | -0.02 | 0.027 | -0.9 | 0.37 |  |  |  |
| ns(temp_lag, df = 4)4 | 0.01 | 0.019 | 0.5 | 0.62 |  |  |  |
| ns(dew_lag, df = 3)1 | -0.02 | 0.011 | -2.1 | 0.04 | 138.1 | 3 | <0.01 |
| ns(dew_lag, df = 3)2 | -0.06 | 0.029 | -1.9 | 0.05 |  |  |  |
| ns(dew_lag, df = 3)3 | -0.16 | 0.016 | 10.1 | <0.01 |  |  |  |
| flu epidemic | 0.05 | 0.006 | 8.9 | <0.01 |  |  |  |
| public holiday | 0.24 | 0.007 | 32.9 | <0.01 |  |  |  |

## Diagnostics

The model diagnostics available to us are dfbetas, which estimate the influence of each observation on the values of each of the regression coefficients, and Martingale residuals, which help us assess the assumption of linearity in the relationship between each of the (continuous) covariates and the log of the risk of presenting to ED. In the input dataset for the coxph function, there are multiple entries for each day (or, more accurately, the people presenting to ED for the specified condition on each day): one observation as a case day and three or four observations as control days (matched on year, month and day of the week to other case days). The default diagnostics from the residuals function will give a value for each observation in the input dataset. To obtain diagnostics aggregated to one value per day, we use the collapse option. For example, to get the overall influence on covariate coefficients of each day we use the following commands: -

dfbetas.col <- residuals(out, type='dfbetas', collapse=indat$date, weighted=T)

where 'out' is the coxph.object returned by the coxph function:

out <- coxph(formla, data= indat, weights= indat$outcome)

and formla is the model that we are fitting:

formla <- reformulate(c(exposure, covariates, 'strata(time)'), response='Surv')

### Influential observations

Figures 1a - 1c show the standardised dfbeta values plotted by date of presentation to ED when modelling ED presentations for respiratory conditions on same-day extreme smoke pollution events (lfs_pm99_lag0), influenza epidemic days (flu), public holidays (pubhol), and natural cubic splines for temperature, humidity (dewpt), lagged temperature (templag) and humidity (dewlag).

There are few days that stand out as being extremely influential. With respect to influence on the smoke event coefficient: there are three or four short periods that have higher influence. This would be expected because there were only 46 smoke event days over the period and these were mainly clustered in three summers (1997/98, 2001/02 and 2003/04). For each variable, we reviewed the data for all days with an absolute value of standardised dfbeta greater than 0.2.

The day with the greatest influence on the smoke event day coefficient was 2 January 1998, when there was a relatively high number of cases (n=162), given the high temperature on the day (27°C) and on the previous 3 days (25°C). Individually, none of these figures stand out as being questionable and so we do not exclude the day from the analysis.

Between 10^th^ and 30th August 2003 there were 5 days with high influence on the temperature coefficients. Four of these days had very high numbers of cases and low same-day or previous 3-day temperatures. Again, in isolation, none of these figures stand out as being particularly extreme and so are not dropped from the analysis.

In summary, the review of days with high influence on the coefficients did not identify any days where the data seemed unreasonable and so no days were excluded from our analysis.


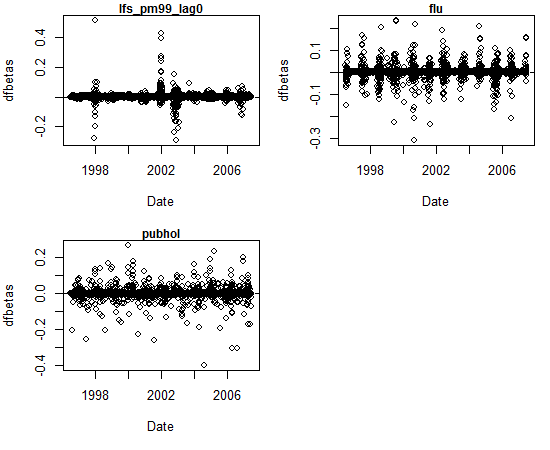


**Figure 1a: Standardised dfbeta values plotted by date of presentation to ED for the binary covariates (smoke event day = ‘lfs_pm99_lag0’, influenza epidemic day = ‘flu’ and public holiday = ‘pubhol’) in the model of all respiratory condition presentations.**
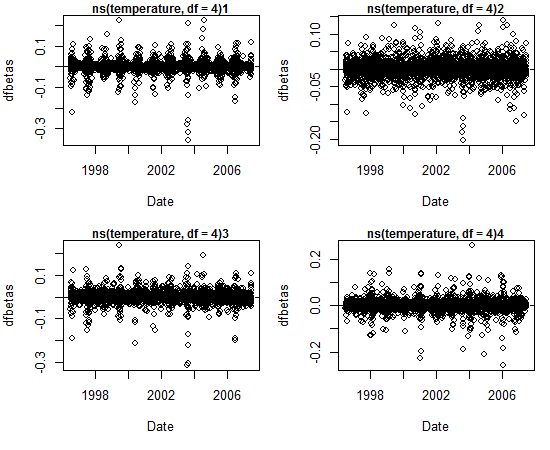

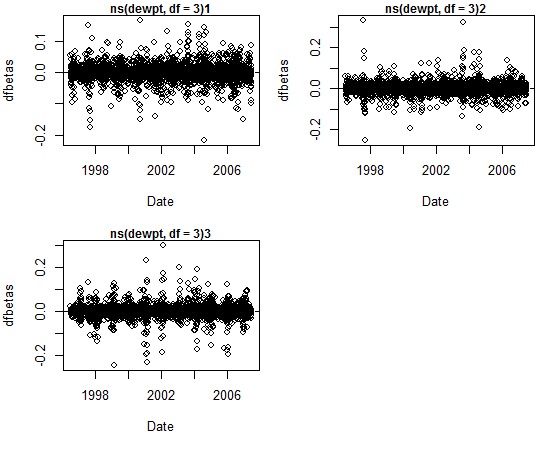


**Figure 1b: Standardised dfbeta values plotted by date of presentation to ED for the same-day temperature and humidity (dewpt) covariates (as spline bases) in the model of all respiratory condition presentations.**


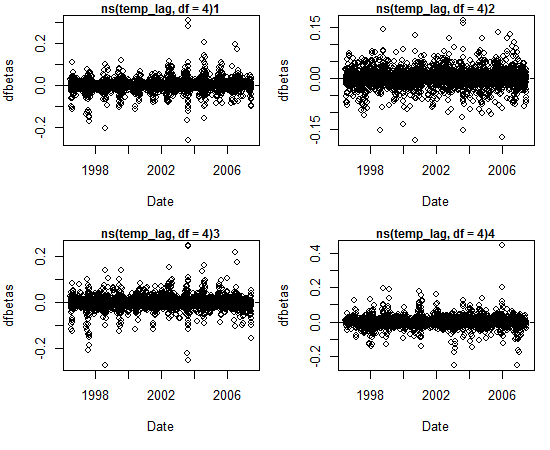


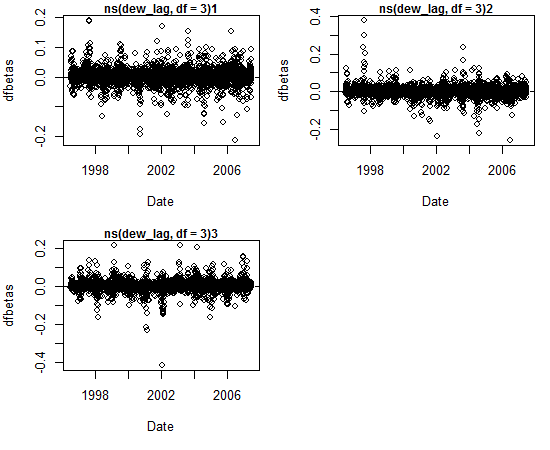


**Figure 1c: Standardised dfbeta values plotted by date of presentation to ED for the lagged temperature (temp_lag) and humidity (dew_lag) covariates (as spline bases) in the model of all respiratory condition presentations.**

### Log-linear relationships

Martingale residuals are used in Cox regression to check that the form of each of the continuous covariates is appropriate, i.e. that the covariate has a linear relationship with the log of the hazard. The plots in figures 2a and 2b are used to check that the natural cubic splines appropriately capture the non-linear relationships between the log of the risk of presentation to ED for respiratory conditions and the four temperature and humidity covariates. The plots are obtained by:-

1. fitting a model that excludes the covariate of interest, obtaining the martingale residuals; and
2. plotting these residuals against the values of the excluded covariate (or its spline basis).

We can be reassured that the form of the covariate is reasonable if we find a linear relationship between the Martingale residuals and the covariate. A lowess smoothing curve is added to each plot to aid in the visual assessment of the linearity.

None of the plots in figures 2a and 2b give serious cause for concern and we conclude that the natural cubic splines for temperature and humidity variables are appropriate.
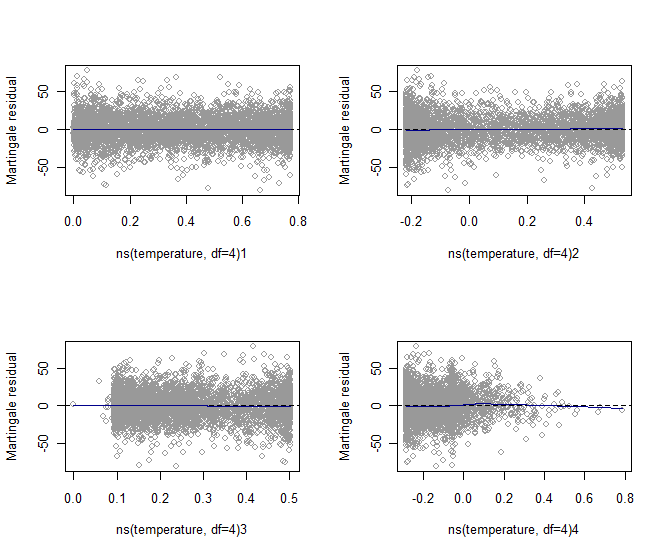


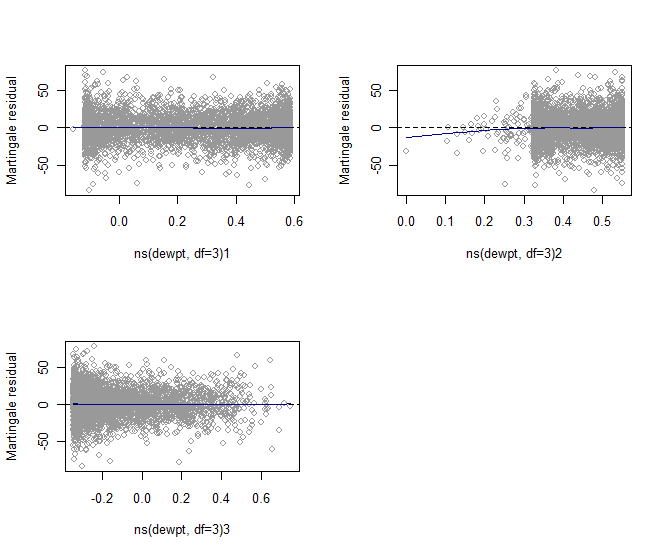


**Figure 2a: Martingale residuals plotted against basis splines for temperature and humidity (dewpt) in the model for all respiratory condition presentations. A dashed line shows martingale=0 and a solid (dark blue) line is a lowess smoother for residual.**


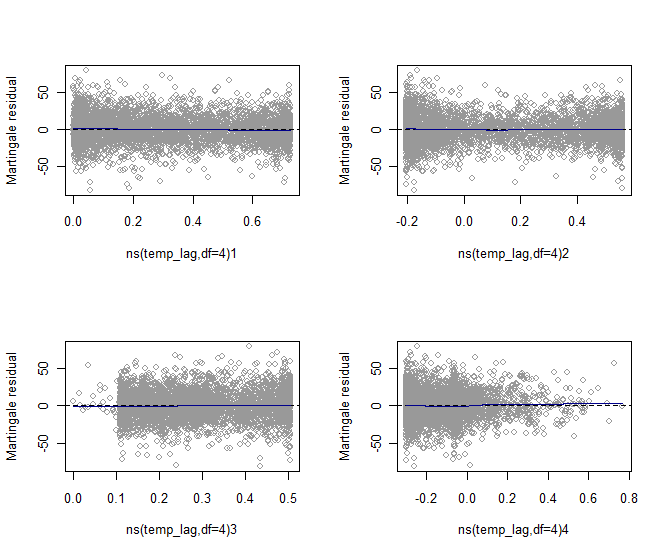


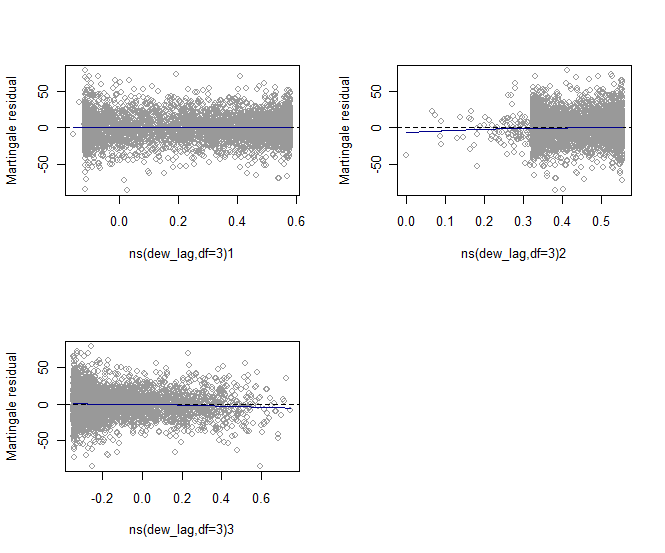


**Figure 2b: Martingale residuals plotted against basis splines for lagged temperature and lagged humidity (dewpt) in the model for all respiratory condition presentations. A dashed line shows martingale=0 and a solid (dark blue) line is a lowess smoother for residual.**

**Sensitivity analysis 1. The influence of using imputed statistical local areas to derive the study population.**

The population of Sydney was 4.06 million at the 2001 census. Participants were identified from the Emergency Department Data Collection (EDDC) maintained by the NSW Ministry of Health for the period 1 July 1996 to 30 June 2007. Records were selected for patients residing in statistical local areas (SLAs) corresponding with the Sydney metropolitan area. We identified a four-month period during 2002 and 2003 when the patient SLA of residence was absent from approximately 80% of records. However, the postcode of residence was available for 98% of these records and we were able to derive the SLA by direct substitution where there was a one-to-one correspondence between the postcode and SLA. In cases where the postcode covered several SLAs, we imputed the SLA of residence by random allocation based on postcode of residence and the proportion of the population in each SLA the postcode covered. After imputation the proportion of records with missing SLAs was reduced to less than one percent. We report all results including the 91,866 ED records with imputed SLA of residence (2.0% of total). Comparing analyses without the imputed SLA demonstrated no appreciable differences in results to those presented in the main paper which included the imputed SLAs. These are presented in Table 3.

**Table 3: Estimated odds ratios (OR) for the associations between smoke event days and presentations to emergency departments by Sydney residents: A comparison of results with and without including the imputed SLA’s in the study population.**

| **Reason for attendance** | **Lag** | **Including imputed SLAs**  **Odds ratio (95% CI)** | **Excluding imputed SLAs**  **Odds ratio (95% CI)** |
| --- | --- | --- | --- |
| All non-trauma presentations | 0 | **1.03 (1.02,1.04)** | **1.03 (1.02,1.04)** |
|  | 1 | **1.02 (1.01-1.03)** | **1.02 (1.01-1.03)** |
|  | 2 | **1.02 (1.01-1.03)** | **1.02 (1.02-1.04)** |
|  | 3 | **1.01 (1.00-1.02)** | **1.01 (1.00-1.03)** |
| All respiratory conditions | 0 | **1.07 (1.04-1.10)** | **1.07 (1.04-1.10)** |
|  | 1 | **1.05 (1.02-1.08)** | **1.05 (1.02-1.08)** |
|  | 2 | 1.00 (0.97-1.03) | 1.00 (0.97-1.03) |
|  | 3 | 1.01 (0.98-1.04) | 1.01 (0.98-1.04) |
| Asthma | 0 | **1.23 (1.15-1.30)** | **1.24 (1.16-1.33)** |
|  | 1 | **1.18 (1.11-1.26)** | **1.19 (1.12-1.27)** |
|  | 2 | **1.14 (1.07-1.22)** | **1.16 (1.09-1.24)** |
|  | 3 | **1.10 (1.03-1.17)** | **1.11 (1.04-1.18)** |
| COPD | 0 | **1.12 (1.02-1.24)** | **1.16 (1.05-1.29)** |
|  | 1 | 1.03 (0.93-1.14) | 1.01 (0.91-1.13) |
|  | 2 | 0.96 (0.87-1.06) | 0.99 (0.89-1.10) |
|  | 3 | 1.03 (0.93-1.14) | 1.03 (0.93-1.15) |
| Pneumonia or acute bronchitis | 0 | 1.02 (0.95-1.10) | 1.02 (0.95-1.10) |
|  | 1 | 1.00 (0.93-1.07) | 1.00 (0.93-1.08) |
|  | 2 | 1.02 (0.95-1.10) | 1.06 (0.98-1.14) |
|  | 3 | 1.00 (0.93-1.07) | 1.02 (0.94-1.10) |
| All cardiovascular conditions | 0 | 1.00 (0.96-1.04) | 1.00 (0.96-1.04) |
|  | 1 | 0.99 (0.95-1.03) | 1.00 (0.96-1.04) |
|  | 2 | 1.03 (0.99-1.06) | **1.04 (1.00-1.08)** |
|  | 3 | 0.99 (0.96-1.03) | 1.00 (0.97-1.04) |
| Ischaemic heart disease | 0 | 0.99 (0.93-1.06) | 0.99 (0.92-1.06) |
|  | 1 | 1.01 (0.95-1.08) | 1.01 (0.93-1.07) |
|  | 2 | **1.07 (1.00-1.15)** | **1.08 (1.00-1.16)** |
|  | 3 | 0.96 (0.90-1.03) | 0.96 (0.89-1.03) |
| Arrhythmias | 0 | 0.97 (0.89-1.06) | 0.98 (0.89-1.07) |
|  | 1 | **0.91 (0.83-0.99)** | **0.92 (0.84-1.00)** |
|  | 2 | 0.93 (0.86-1.02) | 0.93 (0.85-1.02) |
|  | 3 | 0.94 (0.86-1.03) | 0.97 (0.89-1.07) |
| Cerebrovascular diseases | 0 | 0.99 (0.91-1.08) | 0.99 (0.91-1.08) |
|  | 1 | 0.99 (0.91-1.08) | 1.00 (0.90-1.10) |
|  | 2 | 0.97 (0.89-1.06) | 1.00 (0.91-1.10) |
|  | 3 | 1.01 (0.93-1.10) | 1.03 (0.91-1.13) |
| Cardiac failure | 0 | 1.05 (0.95-1.17) | 1.06 (0.94-1.18) |
|  | 1 | 0.95 (0.85-1.05) | 0.95 (0.85-1.07) |
|  | 2 | 1.04 (0.94-1.16) | 1.06 (0.95-1.19) |
|  | 3 | 1.02 (0.91-1.13) | 0.99 (0.89-1.11) |

**Sensitivity analysis 2. The influence of removing adjustment for epidemics of influenza.**

As epidemics of influenza will causes increases hospital attendances for pneumonia and acute bronchitis we believe it was appropriate to adjust for this in the main analysis. However as influenza epidemics usually occur in winter, while fires usually occur at other times of the year, this could have been unnecessary as the main analysis was adjusted for season. In table 4 we present the outputs from the models adjusted, and not adjusted, for influenza epidemics. The coefficients are identical to two decimal places. Small differences in confidence limits at the level of the second decimal place were present in three of 16 models.

Table 4. Estimated odds ratios (OR) for the associations between smoke event days and presentations to emergency departments for Pneumonia or acute bronchitis by age-group. Top panel is adjusted for influenza epidemic periods (as reported in the paper). The bottom panel presents results NOT adjusted for influenza epidemic periods.

| **Adjusted for influenza epidemics** | | | | | | | | | |
| --- | --- | --- | --- | --- | --- | --- | --- | --- | --- |
|  | | **All ages** | | **Under 15** | | **15-64** | | **65 plus** | |
| Lag | | OR | 95% CI | OR | 95% CI | OR | 95% CI | OR | 95% CI |
| 0 | | 1.02 | (0.95-1.10) | 0.96 | (0.85-1.07) | 1.09 | (0.95-1.25) | 1.06 | (0.94-1.19) |
| 1 | | 1.00 | (0.93-1.07) | 0.97 | (0.87-1.09) | 0.89 | (0.78-1.03) | 1.11 | (0.99-1.25) |
| 2 | | 1.02 | (0.95-1.10) | 1.05 | (0.94-1.18) | 0.96 | (0.84-1.11) | 1.04 | (0.92-1.18) |
| 3 | | 1.00 | (0.93-1.07) | 1.01 | (0.90-1.13) | 0.95 | (0.83-1.10) | 1.02 | (0.90-1.16) |
| **NOT adjusted for influenza epidemics** | | | | | | | | | |
| 0 | 1.02 | | (0.95-1.10) | 0.96 | (0.85-1.07) | 1.09 | (0.95-1.26) | 1.06 | (0.94-1.19) |
| 1 | 1.00 | | (0.93-1.07) | 0.97 | (0.87-1.09) | 0.89 | (0.78-1.03) | 1.11 | (0.99-1.25) |
| 2 | 1.02 | | (0.95-1.10) | 1.05 | (0.94-1.17) | 0.96 | (0.84-1.11) | 1.04 | (0.92-1.18) |
| 3 | 1.00 | | (0.93-1.08) | 1.01 | (0.90-1.13) | 0.95 | (0.83-1.10) | 1.03 | (0.91-1.16) |
